# Sustained Delivery of Analgesic and Antimicrobial Agents to Knee Joint by Direct Injections of Electrosprayed Multipharmaceutical-Loaded Nano/Microparticles

**DOI:** 10.3390/polym10080890

**Published:** 2018-08-09

**Authors:** Yung-Heng Hsu, Dave Wei-Chih Chen, Min-Jhan Li, Yi-Hsun Yu, Ying-Chao Chou, Shih-Jung Liu

**Affiliations:** 1 Department of Orthopedic Surgery, Chang Gung Memorial Hospital-Linkou, Tao-Yuan 33305, Taiwan; laurencehsu.hsu@gmail.com (Y.-H.H.); alanyu1007@gmail.com (Y.-H.Y.); enjoycu@ms22.hinet.net (Y.-C.C.); 2 Department of Mechanical Engineering, Chang Gung University, Tao-Yuan 33302, Taiwan; liminjan127001@gmail.com; 3 Department of Orthopedic Surgery, Chang Gung Memorial Hospital-Keelung, Keelung 20401, Taiwan; mr5181@cgmh.org.tw

**Keywords:** electrospraying, biodegradable nano/microparticles, drug delivery, septic arthritis, release characteristics

## Abstract

In this study, we developed biodegradable lidocaine–/vancomycin–/ceftazidime–eluting poly(d,l–lactide–*co*–glycolide) (PLGA) nano/microparticulate carriers using an electrospraying process, and we evaluated the release behaviors of the carriers in knee joints. To prepare the particles, predetermined weight percentages of PLGA, vancomycin, ceftazidime, and lidocaine were dissolved in solvents. The PLGA/antibiotic/lidocaine solutions were then fed into a syringe for electrospraying. After electrospraying, the morphology of the sprayed nano/microparticles was elucidated by scanning electron microscopy (SEM). The in vitro antibiotic/analgesic release characteristics of the nano/microparticles were studied using high-performance liquid chromatography (HPLC). In addition, drug release to the synovial tissues and fluids was studied in vivo by injecting drug-loaded nano/microparticles into the knee joints of rabbits. The biodegradable electrosprayed nano/microparticles released high concentrations of vancomycin/ceftazidime (well above the minimum inhibition concentration) and lidocaine into the knee joints for more than 2 weeks and for over 3 days, respectively. Such results suggest that electrosprayed biodegradable nano/microcarriers could be used for the long-term local delivery of various pharmaceuticals.

## 1. Introduction

Painful swollen joints remain a challenge for physicians and surgeons. The most severe cases are caused by septic arthritis, which occurs in approximately 2–10 out of 100,000 cases per year [1,2,3,4]. Delayed diagnosis without adequate treatment usually leads to permanent and even life-threatening joint damage. Indeed, the mortality rate of monoarticular sepsis is approximately 11% [5].

The most commonly found organisms in septic arthritis include Gram-positive staphylococci and streptococci in various populations, and Gram-negative organisms in older populations [2,6]. Both surgical intervention with arthroscopic irrigation or open arthrotomy to remove purulent material and serial irrigation have been employed as effective treatments for native septic knee arthritis [7]. In 2009, Ravindran et al. demonstrated that medical treatment (closed serial needle aspiration and intravenous antibiotics treatment) has satisfactory therapeutic outcomes for most cases of uncomplicated native septic arthritis [8].

Although debates continue over whether medical treatment or surgical intervention should be the standard of care for native septic arthritis, patients with septic arthritis generally must be admitted to a hospital for advanced assessment and treatment with intravenous antibiotics and/or surgical intervention (i.e., radical joint debridement). Broad-spectrum antibiotics are usually administered after bacterial culture and identification. The consensus for mainstay treatment includes the removal of purulent material combined with a 6-week course of adequate antibiotics, beginning with intravenous drugs followed by oral antibiotics [5]. However, possible alternatives are under study.

Knee injection of pharmaceuticals is commonly employed for the relief of knee discomfort. For that reason, we believe that knee injections of antimicrobial-and analgesic-loaded bioabsorbable nano/microcarriers could be a viable option for the treatment of septic arthritis. The procedure potentially provides high localized concentrations of broad-spectrum pharmaceuticals to knee joints for the treatment of infection and discomfort. Thus, we considered a method for creating such nano/microcarriers.

Electrospraying [9,10] is a simple and versatile electro-hydrodynamic technique for the production of nano/microparticulates [11,12,13] that has been widely employed to produce nano/microcarriers for drug delivery [14,15,16,17]. The process involves the application of a high electric field. When the electrostatic force is sufficiently high, solution at the end of a metallic needle stretches and forms a conical jet that eventually breaks into droplets. As the droplets travel toward the collector, the solvent evaporates and solid particles are collected. Core-shell type nano/microparticles for the encapsulation of susceptible protein-based therapeutic agents such as growth factor can be prepared using coaxial [18,19,20,21] or triaxial [22] electrospraying techniques.

Various studies have been performed on electrospraying and the production of drug-eluting nano/microparticles. Enayati et al. [12] used electrospraying to prepare particles, capsules, and bubbles for biomedical engineering applications. Hao et al. [15] employed electrospraying technology to formulate porous poly(lactic–*co*–glycolic acid) nano/microparticles. They found that the electrospraying method has a considerably shorter production time than the multi-emulsion method. Prabhakaran et al. [14] used electrospraying to fabricate metronidazole-loaded PLGA particles that provide a minimum of 41 days of sustained drug release in phosphate-buffered saline (PBS). Songsurang et al. [16] adopted electrospraying for the preparation of doxorubicin-loaded chitosan nanoparticles. They proposed that high encapsulation efficiency for doxorubicin-loaded nanoparticles was attainable. Malik et al. [17] reported a formulation recipe for generating spherical poly(lactic–*co*–glycolic acid) microspheres. Their technique employed electrospraying coupled with a novel thermally-induced phase separation process combined with a tailored liquid nitrogen collection scheme for the production of controlled-release medication.

Among the various polymers available for the formulation of local release systems, poly(d,l–lactide–*co*–glycolide) (PLGA) is one of the most thoroughly researched biodegradable materials due to its favorable properties including its highly controlled and consistent degradation characteristics and outstanding reproducible mechanical and physical properties [23,24].

The present study used electrospraying to develop biodegradable lidocaine–/vancomycin–/ceftazidime–eluting PLGA nano/microcarriers, which were subsequently evaluated for in vitro and in vivo release behaviors. Vancomycin was chosen because it is widely used for the treatment of infections complicated by Gram-positive methicillin-resistant *Staphylococcus aureus*. Meanwhile, ceftazidime exhibits strong activity against susceptible Gram-negative bacteria. Lidocaine is a cardiac depressant used as an antiarrhythmic agent [25], but it is also used as a local anesthetic. 

## 2. Materials and Methods

To prepare the biodegradable drug-eluting particles, predetermined weight percentages of PLGA, vancomycin, ceftazidime, and lidocaine were dissolved in solvents. The PLGA/antibiotic/lidocaine solutions were then fed into a syringe for electrospraying. After electrospraying, the morphology of the sprayed microparticles was examined by scanning electron microscopy (SEM). In addition, the in vitro microparticle antibiotic/analgesic release characteristics were elucidated via high-performance liquid chromatography (HPLC). To characterize in vivo medication release to synovial tissues and fluids, drug-loaded nano/microparticles were injected into the knee joints of New Zealand white rabbits.

### 2.1. Fabrication of Biodegradable Drug-Eluting Microparticles

All materials utilized in this study, including PLGAs (50:50 with a molecular weight of 33,000 Da, and 75:25 with a molecular weight of 70,000 Da), vancomycin hydrochloride, ceftazidime hydrate, and lidocaine hydrochloride, were purchased from Sigma-Aldrich (St. Louis, MO, USA). Three types of solvents, namely dichloromethane (DCM), chloroform (CHF), and 1,1,1,3,3,3-hexafluoro-2-propanol (HFIP; Sigma-Aldrich), were used in the experiments.

**Figure 1:**
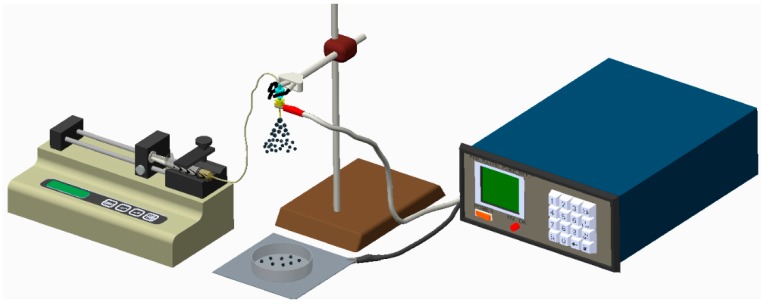
Schematic of the experimental setup to electrospray the drug-eluting nano/microcarriers.

Antibiotic–/analgesic–eluting nano/microparticles were prepared using the electrospraying device shown in Figure 1, which consisted of a syringe and needle (internal diameter of 0.60 mm), ground electrode, collector, and high voltage supply. PLGA and pharmaceuticals (360 mg PLGA, 60 mg vancomycin, 60 mg ceftazidime, and 60 mg lidocaine) [25] were first dissolved in 6 mL of solvent and mixed with a magnetic stirrer for 12 h. Three different types of solvents were employed to determine their feasibility for electrospraying. During electrospraying, the solution was transported utilizing a syringe pump that possessed a flow rate of 0.6 mL per hour. The journey distance between the needle and the collection was 20 cm.

### 2.2. Scanning Electron Microscopy

The morphology of electrosprayed nano/microparticles was observed on a field emission scanning electron microscope (FESEM; JEOL Model JSM-7500F, Tokyo, Japan) after coating with gold.

### 2.3. Infrared Spectrometry

Fourier transform infrared (FTIR) spectrometry was employed to examine the spectra of pure PLGA nano/microparticles and drug-loaded PLGA nano/microparticles. The FTIR analysis was conducted on a Nicolet iS5 spectrometer (Thermo Fisher Scientific, Waltham, MA, USA) at a resolution of 4 cm^−1^ (32 scans). Electrosprayed particles were pressed as KBr discs, and spectra were recorded over the range of 400–4000 cm^−1^.

### 2.4. In Vitro Liberation of Antibiotics and Analgesic

The in vitro release of antibiotics and analgesics from the nano/microparticles was evaluated by an elution method. Particles of predetermined weights were added to the test tubes. Each tube contained 1 mL PBS and was incubated at 37 °C for 24 h. The eluent was then collected and replaced by 1 mL new PBS. This elution process was duplicated for 30 days. The concentrations of lidocaine and antibiotics in the collected solutions were quantified by HPLC, which was conducted on a Hitachi L-2200 Multisolvent Delivery System.

A Symmetry C_8_ column (3.9 cm × 150 mm) was used to characterize vancomycin and ceftazidime (Waters, Milford, MA, USA), while an Atlantis dC18, 4.6 cm × 150 mm column (Waters Corp., Milford, MA, USA) was employed to evaluate lidocaine. The mobile phase used to separate vancomycin contained 0.01 mol heptanesulfonic acid (Fisher Scientific, Loughborough, UK) and acetonitrile (85/15, *v*/*v*; Mallinckrodt, St. Louis, MO, USA). The absorbance wavelength and flow rate were 280 nm and 1.4 mL/min, respectively. The mobile phase utilized for ceftazidime was composed of methanol and PBS (5 mM, pH 7.5) with a volumetric ratio of 10:90. The absorbance was monitored at 254 nm with a flow rate of 0.6 mL/min. The mobile phase used to quantify lidocaine consisted of ammonium formate and methanol (20/80, *v*/*v*; Sigma-Aldrich). The absorbance wavelength was set at 210 nm, while the flow rate was maintained at 1.0 mL/min. All experiments were performed in triplicate (*N* = 3).

### 2.5. In Vivo Study

**Figure 2:**
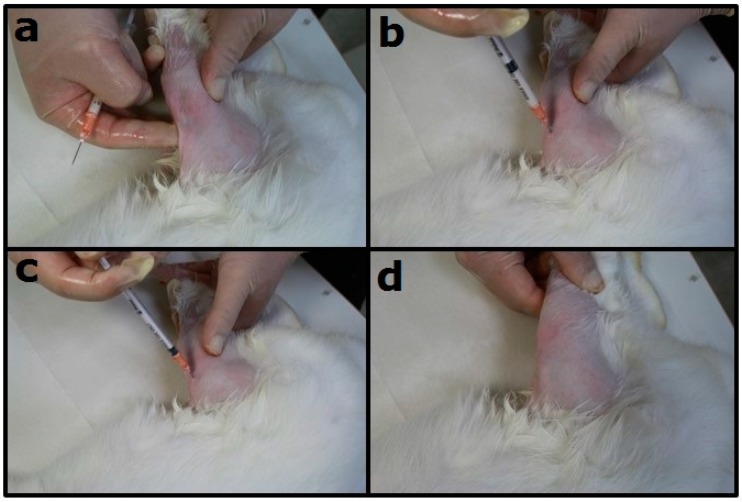
Knee injection with biodegradable microparticles to a New Zealand white rabbit. (**a**) The surgical site was shaved and the aseptic procedure was performed at the injection site; (**b**) the supra-patella pouch of the knee joint of rabbit was identified and penetrated with a needle; (**c**) 1 mL phosphate-buffered solution (PBS) + 200 mg drug-loaded nano/microparticles was injected; (**d**) free range of motion after injection was confirmed.

Six New Zealand white rabbits weighing 2.8–3.2 kg were enrolled in the experiment. All animal-related procedures were approved by an institutional animal care and use committee (IACUC Approval No. CGU106-058). All animals received general anesthesia by inhaling isoflurane through a vaporizer (Matrx, Pompano Beach, FL, USA) in a plastic box (40 cm × 20 cm × 28 cm). Anesthesia was maintained during the entire injected procedure via mask inhalation of isoflurane. After the rabbits were sedated, the injected sites were depilated, washed with soft soap, and disinfected with 70% ethanol directly before the injection procedure. The posterior part of the animals was covered with a sterile blanket. The test solution (1 mL PBS + 200 mg drug-loaded nano/microparticles) was then directly injected into their bilateral knee joints (Figure 2).

**Figure 3:**
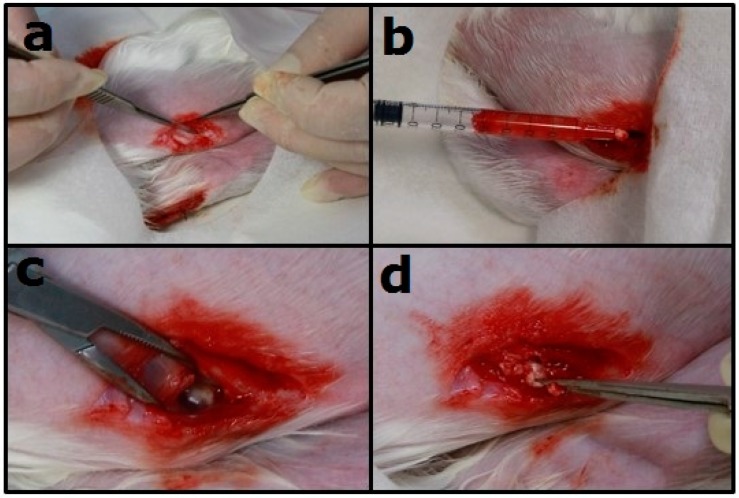
Sampling the specimen from the knee joint of New Zealand white rabbits. (**a**) The knee joint of the rabbit was opened; (**b**) the synovial fluid was sampled using a syringe; (**c**) the wound was opened; (**d**) the residual drug-eluting nano/microparticles could be seen.

The vivo drug concentrations were determined by sampling specimens (synovial tissue and synovial fluid) on Days 1, 3, 7, and 14 after the injected procedure. Surgery of open biopsy was completed to obtain the specimens (Figure 3). The vancomycin, ceftazidime, and lidocaine concentrations of the specimens were determined by the same HPLC assay described in Section 2.4.

## 3. Results

### 3.1. Characterization of Electrosprayed Particles

**Figure 4:**
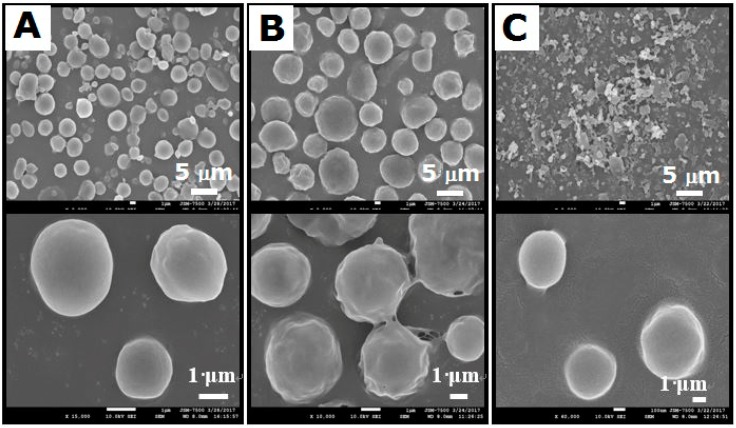
Drug-eluting 50:50 PLGA nano/microparticles fabricated using different solvents: (**A**) dichloromethane (DCM); (**B**) chloroform (CHF); (**C**) 1,1,1,3,3,3-hexafluoro-2-propanol (HFIP).

Three different types of solvents (i.e., DCM, CHF, and HFIP) were used to prepare the drug-eluting particles (50:50 PLGAs). Figure 4 shows SEM images of electrosprayed particles prepared with different solvents. Those made with DCM and CHF ranged from 2–5 μm to 5–10 μm, while electrosprayed particles prepared with HFIP were approximately 500 nm. Because the particles prepared with CHF exhibited a more uniform size and geometric distribution compared to those of other solvents, CHF was chosen for use in subsequent in vivo tests.

**Figure 5:**
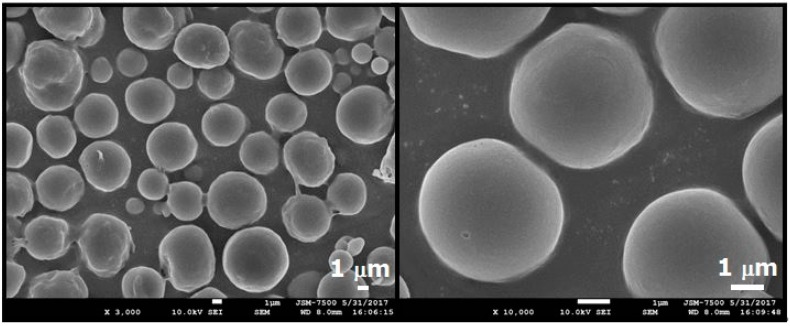
Drug-eluting 75:25 PLGA nano/microparticles (DCM was used as the solvent).

The influence of polymer types on the particles was also investigated. Figure 5 shows electrosprayed drug-loaded 75:25 PLGA particles (with DCM as the solvent). The size of the particles was approximately 10 μm, which is a little larger than that of the 50:50 PLGA microparticles.

**Figure 6:**
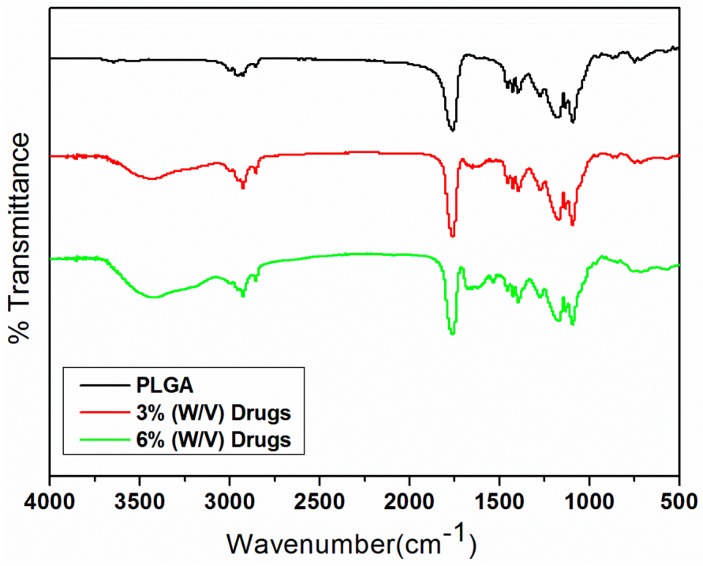
FTIR spectra of electrosprayed nano/microparticles. Abbreviations: FTIR, Fourier transform infrared; PLGA, poly(d,l)–lactide–*co*–glycolide.

Figure 6 displays the measured spectra of virgin PLGA microparticles and antibiotic/analgesic-loaded PLGA microparticles. The new absorption peak at 3200~3500 cm^−1^ could be attributable to the N–H bonds of vancomycin, ceftazidime, and lidocaine [26,27,28]. The absorption at 1725 cm^−1^ corresponded to C=O bonds, primarily due to the addition of antibiotics and analgesic; the absorbance peak near 1300 cm^−1^ may be the result of C–O bond enhancement in loaded pharmaceuticals. The FTIR spectra illustrates that the antibiotics and analgesic were successfully incorporated into PLGA microparticles.

### 3.2. In Vitro Release of Drug-Eluting Nano/Microparticles

**Figure 7:**
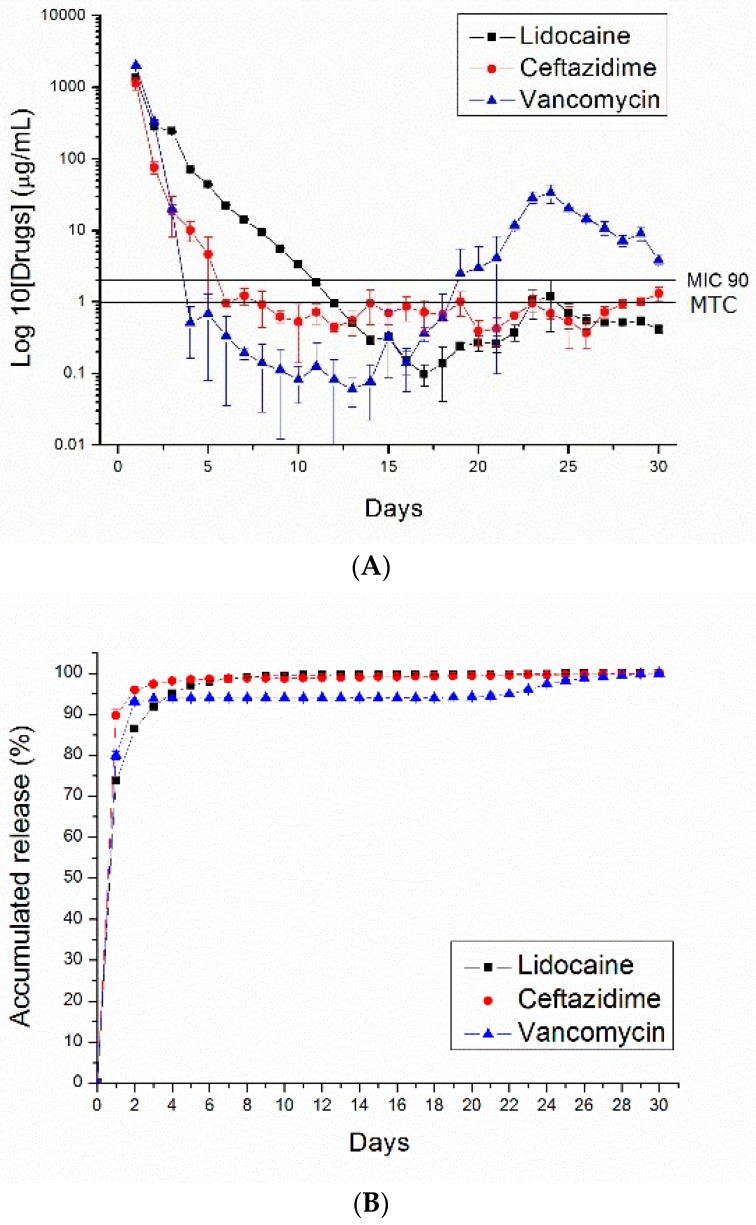
PLGA (50:50)—(**A**) daily and (**B**) accumulated release curves of pharmaceuticals from 50 mg PLGA nano/microparticle-loaded buffered solution.

**Figure 8:**
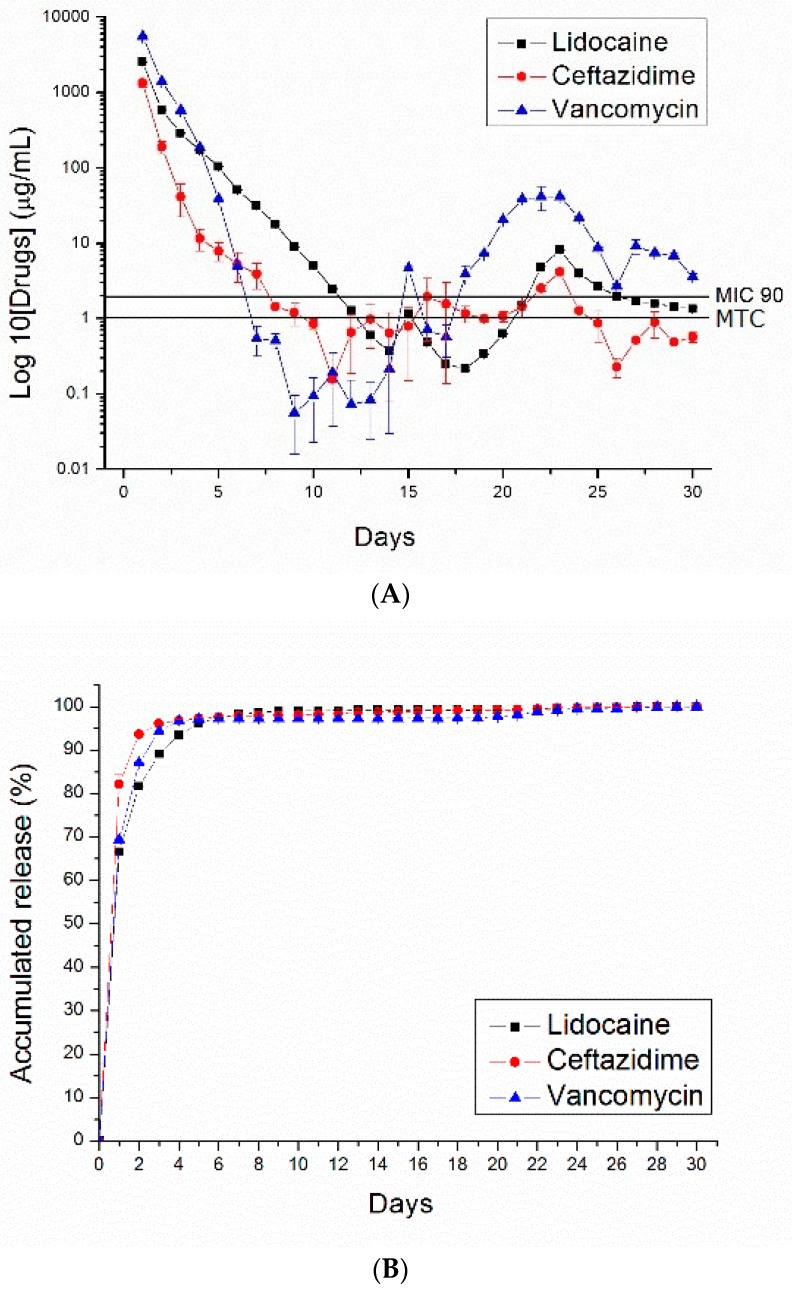
PLGA (50:50)—(**A**) daily and (**B**) accumulated release curves of pharmaceuticals from 100 mg PLGA nano/microparticle-loaded buffered solution.

**Figure 9:**
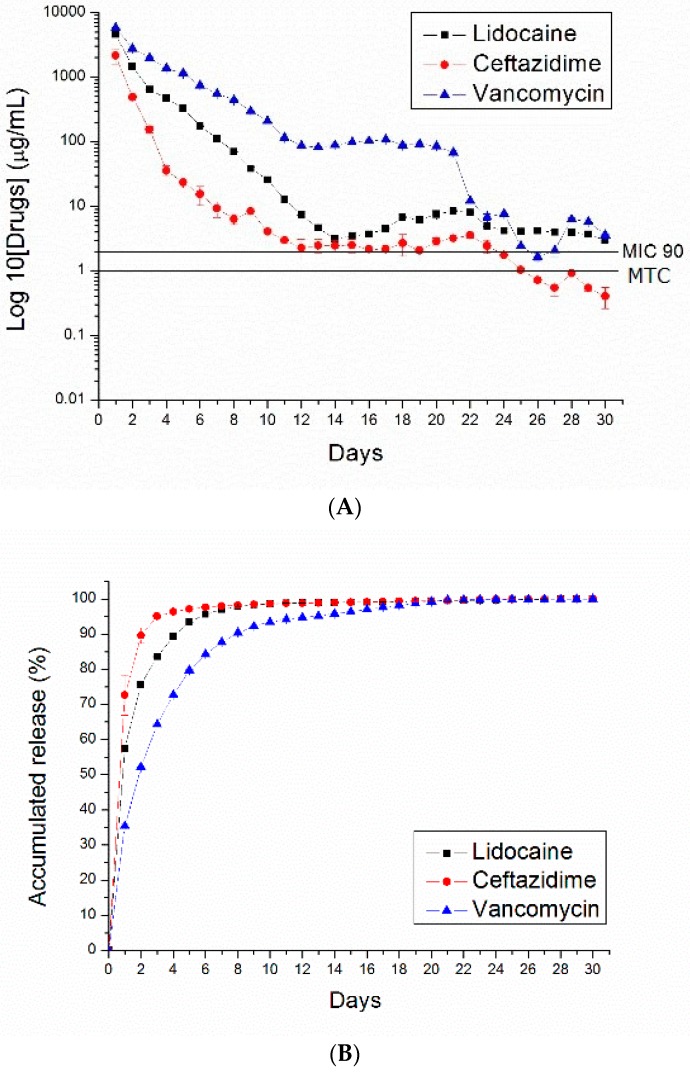
PLGA (50:50)—(**A**) daily and (**B**) accumulated release curves of pharmaceuticals from 200 mg PLGA nano/microparticle-loaded buffered solution.

**Figure 10:**
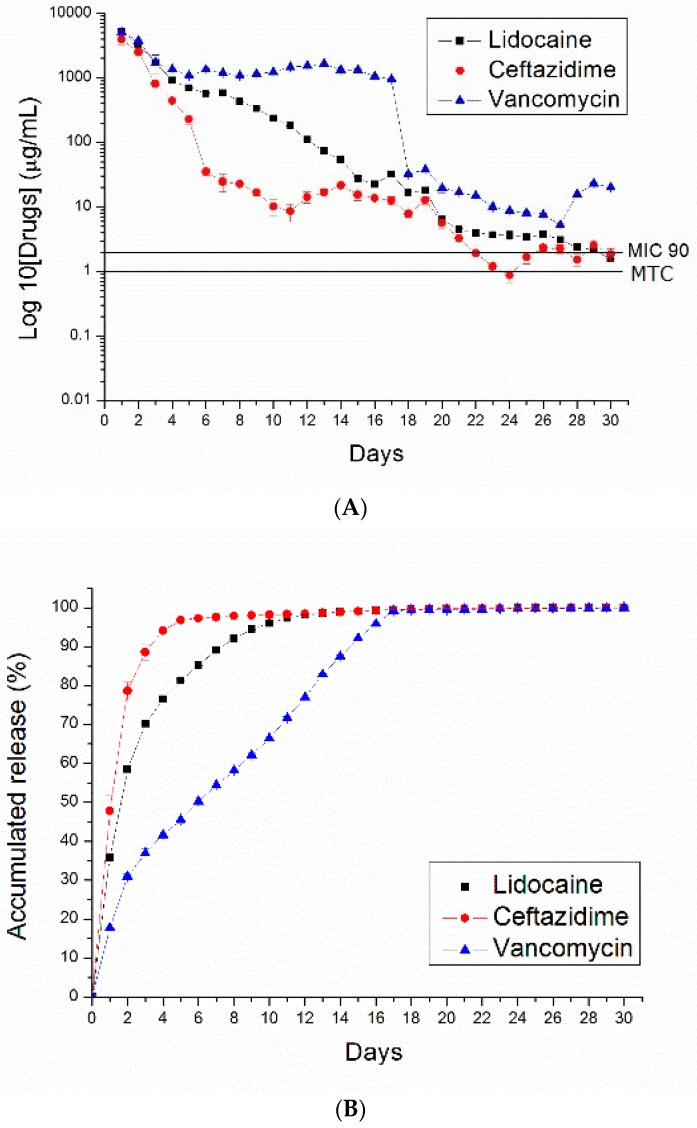
PLGA (50:50)—(**A**) daily and (**B**) accumulated release curves of pharmaceuticals from 400 mg PLGA nano/microparticle-loaded buffered solution.

Figure 7, Figure 8, Figure 9 and Figure 10 show the measured daily and accumulated release curves of vancomycin, ceftazidime, and lidocaine from the electrosprayed drug-eluting nano/microparticles of varying particle weight loadings (50, 100, 200, 400 mg) immersed in 1 mL PBS. Triphasic and biphasic curve patterns were observed for antibiotics and lidocaine, respectively. The initial burst release pattern was observed within days for all pharmaceuticals at all particle weight loads. The second peak release of pharmaceuticals was observed on Days 24, 23, 22, and 19 for particle loads of 50, 100, 200, and 400 mg, respectively. The third peak release of antibiotics was noted on Day 28.

The 50 and 100 mg nano/microparticle-loaded solutions exhibited similar release patterns, characterized by an initially high release on Day 1 and a second peak release after 20 days, followed by a more gradual and sustained release of the drugs. As drug loads increased to 200 and 400 mg, the second drug release peaks gradually declined and the total period of sustainable drug release increased. For example, the 50 mg drug-loaded nano/microparticle solution released high concentrations of vancomycin and ceftazidime, well above the minimum inhibition concentration 90% (MIC_90_), for only 3 and 5 days, respectively, whereas the 400 mg drug-loaded solution liberated high antibiotic strengths (above MIC_90_) for 30 and 21 days, respectively.

**Figure 11:**
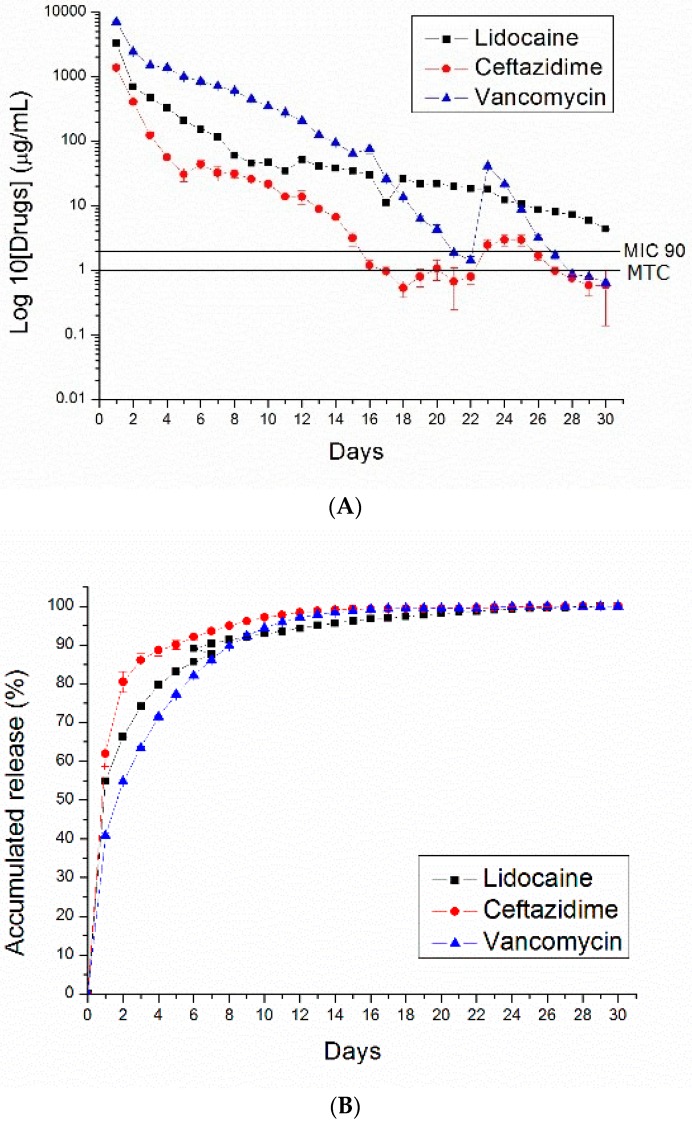
PLGA (75:25)-(**A**) daily and (**B**) accumulated release curves of pharmaceuticals from 200 mg PLGA nano/microparticle-loaded buffered solution.

For comparison, drug-loaded 75:25 PLGA nano/microparticles were also prepared. Figure 11 shows the daily and accumulated release curves of vancomycin, ceftazidime, and lidocaine from electrosprayed drug-eluting 75:25 PLGA nano/microparticles (with a particle loading of 200 mg). Compared to 50:50 PLGA (Figure 9), 75:25 PLGA nano/microparticles exhibited a slower drug release rate. This is probably due to the fact that 75:25 PLGA has a greater molecular weight than 50:50 PLGA. Degradation and drug release were reduced accordingly.

### 3.3. In Vivo Release

**Figure 12:**
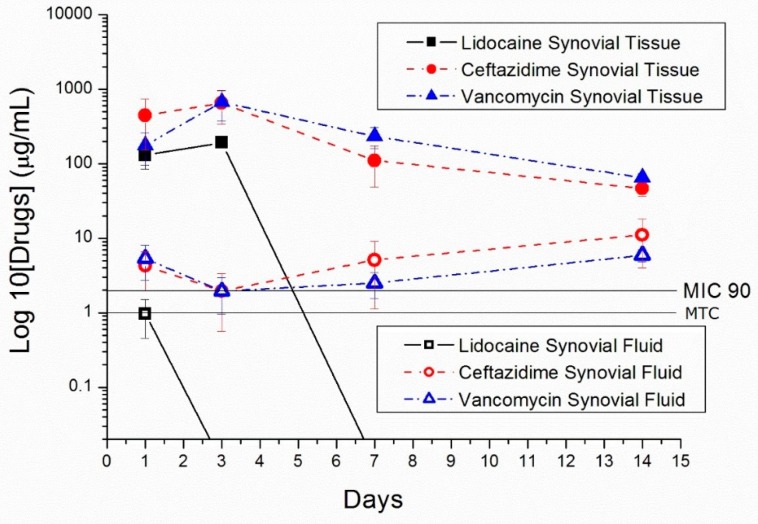
In vivo drug release curves of nano/microparticles in New Zealand white rabbits.

The in vivo release of nano/microparticles in the knee joints of rabbits was characterized. Figure 12 displays the measured drug concentrations in synovial fluid and synovial tissue. The results demonstrate that drug-eluting nano/microparticles can provide a sustained release of high concentrations of vancomycin/ceftazidime (above MIC_90_) and lidocaine (above the minimum therapeutic concentration, MTC [29]) for more than 14 days and 3 days, respectively.

## 4. Discussion

Although both osteomyelitis and periprosthetic joint infections are commonly treated with vancomycin and ceftazidime combined with bone cement consisting of poly(methyl methacrylate) (PMMA) [30,31], PMMA is nonbiodegradable; it requires surgical insertion and removal. Thus, such treatment is not appropriate for native septic arthritis because the presence of foreign bodies may reduce the motion of knee joints. In contrast, the much small size of biodegradable nano/microparticles allows them to be easily injected into knee joints. They have less influence on the knees’ range of motion than do larger particles, and their biodegradable nature eliminates the need for surgical removal.

Debates have continued during the past years over what standard of care is needed to treat native septic arthritis, by medical treatment or surgical intervention. Both surgical intervention with arthroscopic irrigation or open arthrotomy to remove purulent material and serial irrigation have been employed as an effective treatment for native septic knee arthritis [7]. Vinod et al. demonstrated the satisfactory therapeutic outcomes of medical treatment (closed serial needle aspiration and intravenous antibiotics treatment) for most uncomplicated cases of native septic arthritis [8]. However, no strong evidence is available and even no randomized clinical trial has been completed regarding the appropriate duration of antibiotics usage in septic arthritis treatment. The consensus milestone therapy includes the removal of purulent material and the subsequent use of adequate antibiotics for 6 weeks [5]. This study reports the successful development of multidrug-loaded PLGA nano/microparticles using electrospraying technology. The biodegradable drug-eluting nano/microparticles released high concentrations of antibiotics for over 2 weeks in vivo, which is advantageous for infection control at the knee joint. Based on the literature, the current work is the first study that explores the sustained release of high and local antibiotic concentrations in vivo at the native septic arthritis area with the injection of drug-eluting nano/microparticles. Furthermore, the nano/microparticles also released lidocaine at the target site for more than three days, offering the benefit of early-stage pain relief at the joint. The experimental results demonstrated that most of the pharmaceuticals were absorbed by the surrounding tissues. Moreover, serial injections of various doses of nano/microparticles at the knee may offer a theoretically staged delivery of various broad-spectrum antibiotics to the target site, if the initial antibiotics treatment was not sensitive to the identified bacteria.

PLGA is among the most suitable biodegradable polymeric materials used for the fabrication of drug delivery devices and tissue engineering [32]. The material is biocompatible and biodegradable, exhibits a wide range of erosion times, and possesses tunable mechanical properties. Most importantly, PLGA is an FDA-approved polymer that has been extensively studied for the controlled release of small molecule drugs, proteins, and other macromolecules. Herein, we fabricated biodegradable drug-eluting PLGA nano/microparticles loaded with vancomycin, ceftazidime, and lidocaine.

Drug release from a biodegradable carrier can be generally divided into three stages, including a primary blast, a diffusion-dominated elution, and a degradation-dominated release. Meanwhile, the clustering effect of nano/microparticles also contributes to and affects release behavior.

After the electrospraying process, most drugs are dispersed into the volume of the nano/microparticles, but a few pharmaceutical compounds located on the particles’ surface may lead to an initial drug release burst. All pharmaceuticals used in this study were water soluble. However, the octanol-water ratios (log P) (PubChem Compound Database) of vancomycin, lidocaine, and ceftazidime are 4.21, 2.84, and 0.53, respectively. Vancomycin has relatively non-water-soluble characteristics compared to those of lidocaine and ceftazidime; thus, it exhibited the fastest and greatest amount of release during the first stage. Meanwhile, vancomycin had the lowest percentage of accumulated release during the first stage, suggesting that most of the vancomycin was located inside the nano/microparticles.

Following the initial burst, the drug release profiles were not only controlled by diffusion, but also by polymer degradation. A relatively constant decline in the release rate of the antibiotics and analgesic was thus observed. As the number of nano/microparticles increased, clustering occurred, thus reducing the total surface area of the particles. The release rates of vancomycin, ceftazidime, and lidocaine decreased accordingly. Increased nano/microparticle-loading also raised the total amount of incorporated pharmaceuticals, which prolonged the effective drug release period. The third peak release behavior was only noted in the curves of antibiotics (all drug loads) after approximately 28 days. We believe that PLGA nano/microparticles produced biocompatible hydrolysis products (lactic and glycolic acids) upon biodegradation, liberating antibiotics and analgesic from within the nano/microparticulate carriers with drug release patterns similar to those observed on Day 1.

Substances with smaller molecular weights theoretically exhibit more rapid release rates during the diffusion period than those with larger molecular weights. In this study, the molecular weights of lidocaine, ceftazidime, and vancomycin were 323.34, 546.57, and 1449.27, respectively (PubChem Compound Database). Most of the lidocaine leached out of the nano/microparticles during the diffusion period over the first few days.

Vancomycin is notorious for renal toxicity. There is always a safety concern regarding vancomycin blood levels during the therapeutic period when high-dose drugs are administered systemically [31,33]. Our previous study on New Zealand white rabbits demonstrated that blood creatinine levels remain normal during the experiment when vancomycin combined with a biodegradable nanofibrous carrier is administered locally at a fracture site [34]. The experimental results in this study further suggested that the drug absorption rate of the tissues surrounding the drug-eluting nano/microparticles is high. This would provide advantages in terms of transporting high anti-microbial drug levels to the target site with minimized systemic side influence.

It is generally accepted that the sustained release of high local antibiotic concentrations contributes to infection control. Results of the current study demonstrated that biodegradable drug-eluting nano/microparticles could release high concentrations of vancomycin/ceftazidime (well above the MIC_90_) and lidocaine for more than 14 days and 3 days, respectively. FTIR analysis also suggested that the pharmaceuticals loaded into nano/microparticles were not damaged by the electrospraying process. Thus, we believe that electrospray technology will enable the manufacture of multidrug-eluting nano/microparticles that will benefit the treatment of various diseases by providing sustained local drug delivery via particle injection.

## 5. Conclusions

This study successfully used electrospray technology to develop multidrug-loaded PLGA nano/microparticles. It is the first in vivo study to explore the local injection of drug-eluting nano/microparticles into knee joints, an area that can be affected by native septic arthritis, in order to achieve the sustained release of high concentrations of antibiotics. Indeed, the biodegradable drug-eluting nano/microparticles released high concentrations of antibiotics for over 2 weeks in vivo, and the drug absorption rate of the surrounding tissues was high, which is advantageous for the control of infection in knee joints. The nano/microparticles also released lidocaine at the target sites for more than 3 days, conferring early-stage pain relief. 

In addition to integrating infection-control and pain relief strategies, our novel drug-eluting carriers have other practical clinical implications. For instance, serial injection of various doses of nano/microparticles at the knee might theoretically provide staged delivery of various broad-spectrum antibiotics to the target site, which is potentially useful in cases where the identified bacteria is not sensitive to initial antibiotic treatment. For this reason, further research on multidrug-loaded PLGA nano/microparticles is warranted.
